# The Classic Approach to Diagnosis of Vulvovaginitis: A Critical Analysis

**DOI:** 10.1155/S1064744901000187

**Published:** 2001

**Authors:** Jacob Bornstein, Yaniv Lakovsky, Idit Lavi, Amiram Bar-Am, Haim Abramovici

**Affiliations:** 1 Department of Obstetrics and Gynecology Carmel Medical Center and the Rappaport Faculty of Medicine Hatechnion University 7 Michal Street Haifa 34362 Israel; 2 Department of Community Medicine and Epidemiology Carmel Medical Center and the Rappaport Faculty of Medicine Hatechnion University Haifa Israel; 3 Department of Obstetrics and Gynecology Lis Maternity Hospital and the Sackler Faculty of Medicine Tel Aviv University Tel Aviv Israel

## Abstract

*Objective:* To correlate the symptoms, signs and clinical diagnosis in women with vaginal discharge, based on the combined weight of the character of the vaginal discharge and bedside tests, with the laboratory diagnosis.

*Methods:* Women presenting consecutively to the women's health center with vaginal discharge were interviewed and examined for assessment of the quantity and color of the discharge. One drop of the material was then examined for pH and the whiff test was done; a wet mount in saline and in 10% KOH was examined microscopically. The clinical diagnosis was based on the results of these assessments. Gram stain and cultures of the discharge were sent to the microbiology laboratory.

*Results:* One hundred and fifty-threewomen with vaginal discharge with a clinical diagnosis of vulvovaginitis participated in the study. Fifty-five (35.9%) had normal flora and the other 98 (64.1%) had true infectious vulvovaginitis (k agreement = 18%). According to the laboratory, the principal infectious micro-organism causing the vulvovaginitis was *Candida* species. *Candida* infection was associated with pH levels of less than 4.5 (*p* < 0.0001, odds ratio = 4.74, 95% confidence interval: 2.35–9.5, positive predictive value 68.4%). The whiff test was positive in only a small percentage of bacterial vaginosis (BV) (*p* = not significant (NS)). Clue cells were documented in 53.3% of patients with a laboratory diagnosis of BV (*p* < 0.02, positive predictive value 26.7%).

*Conclusions:* The current approach to the diagnosis of vulvovaginitis should be further studied. The classical and time-consuming assessments were shown not to be reliable diagnostic measures.


Infect Dis Obstet Gynecol 2001;9:105–111

### The classic approach to diagnosis of vulvovaginitis: a critical analysis

**Jacob Bornstein[1], Yaniv Lakovsky[1], Idit Lavi[2], Amiram Bar-Am[3] and Haim Abramovici[1]**

[1]*Department of Obstetrics and Gynecology, and* [2]*Department of Community Medicine and Epidemiology, Carmel Medical Center and the Rappaport Faculty of Medicine, Hatechnion University, Haifa, Israel*
[3]*Department of Obstetrics and Gynecology, Lis Maternity Hospital and the Sackler Faculty of Medicine, Tel Aviv University, Tel Aviv, Israel*

***Objective:*** To correlate the symptoms, signs and clinical diagnosis in women with vaginal discharge, based on the combined weight of the character of the vaginal discharge and bedside tests, with the laboratory diagnosis.
***Methods:*** Women presenting consecutively to the women's health center with vaginal discharge were interviewed and examined for assessment of the quantity and color of the discharge. One drop of the material was then examined for pH and the whiff test was done; a wet mount in saline and in 10% KOH was examined microscopically. The clinical diagnosis was based on the results of these assessments. Gram stain and cultures of the discharge were sent to the microbiology laboratory.
***Results:*** One hundred and fifty-three women with vaginal discharge with a clinical diagnosis of vulvovaginitis participated in the study. Fifty-five (35.9%) had normal flora and the other 98 (64.1%) had true infectious vulvovaginitis ($\kappa$ agreement = 18%). According to the laboratory, the principal infectious micro-organism causing the vulvovaginitis was *Candida* species. *Candida* infection was associated with pH levels of less than 4.5 ($p < 0.0001$, odds ratio = 4.74, 95% confidence interval: 2.35–9.5, positive predictive value 68.4%). The whiff test was positive in only a small percentage of bacterial vaginosis (BV) ($p$ = not significant (NS)). Clue cells were documented in 53.3% of patients with a laboratory diagnosis of BV ($p < 0.02$, positive predictive value 26.7%).
***Conclusions:*** The current approach to the diagnosis of vulvovaginitis should be further studied. The classical and time-consuming assessments were shown not to be reliable diagnostic measures.

Key words: VULVOVAGINITIS, *CANDIDA*, BACTERIAL VAGINOSIS, *TRICHOMONAS VAGINALIS*

The diagnosis of vulvovaginitis (VV) has been a matter of controversy. Many texts warn the practicing gynecologist to avoid diagnosing VV based exclusively on the patient's presenting symptoms of vaginal discharge with or without pruritus or on speculum examination, but rather to rely on the 'bedside' tests – pH, whiff test and wet mount microscopy[1–3]. However, in practice many gynecologists make the diagnosis of VV without resorting to any of the recommended tests. Another controversy surrounds the question of whether a culture of the discharge is obligatory in order to diagnose VV. Culture is considered the 'gold standard' in the diagnosis of vulvovaginal candidiasis (VVC)[2,4] and for *Trichomonas vaginalis* vaginitis[5]. While some authors are convinced that

Correspondence to: Jacob Bornstein, MD, Department of Obstetrics and Gynecology, Carmel Medical Center, 7 Michal Street, Haifa 34362, Israel. E-mail: mdjacb@tx.technion.ac.il in most cases the bedside tests are sufficient to diagnose all types of VV, others advocate performing a culture in most cases in order to make the proper diagnosis[4,5]. But is that necessarily so? Are the bedside tests sensitive and specific, or are they a waste of money and time? Is culture necessary or redundant?

The present study correlates each of the symptoms and signs seen in women with vaginal discharge, as well as the clinical diagnosis which was based on the combined weight of the symptoms and findings, with the laboratory diagnosis, i.e. the culture result or the Gram stain finding.

### SUBJECTS AND METHODS

Consecutive women presenting to the women's health center between January 1993 and December 1998, in a medium-income suburb, with troublesome vaginal discharge, with or without vulvar pruritus or abnormal odor, were considered for enrollment to the study. Only those who had not received treatment for vaginitis during the previous month were enrolled. All data were documented on a special form. The local and national Helsinki review boards approved the study and informed consent was obtained from patients appropriate for enrollment to the study. Initially the physician questioned the woman about the symptoms of vaginitis, then examined her for assessment of the quantity and color of the vaginal discharge. A drop of the discharge was then examined for pH, and a whiff test was done. This was followed by microscopic examination of a wet mount, prepared by immersing one drop of discharge in normal saline and one drop in 10% KOH solution. Based on the results of these tests a clinical diagnosis was established (Table 1). A Gram stain of the discharge was sent to the microbiological laboratory for interpretation, and cultures of the discharge taken. Definitive isolation of *Candida* strains was also available. The vaginal discharge was cultured on (1) blood agar base + 5% human blood; (2) McKonkey agar for isolation and definition of *Candida* species; (3) immediate culture into a glass tube containing modified Diamond agar[6], for isolation of *Trichomonas*. An endocervical smear was obtained for the detection of gonorrhea on Thayer Martin chocolate agar and, in a different tube, for the detection of *Chlamydia* by the Gen-probe method. All microbiological isolations were carried out in the hospital laboratory, which uses quality control measures and is National External Quality Assessment Schemes (NEQAS) approved.

For the purpose of the study the diagnosis made by the physician was referred to as the 'clinical diagnosis' while the diagnosis based on the laboratory cultures and Gram stain was referred to as the 'laboratory diagnosis'. Table 1 introduces the parameters used to establish the clinical and laboratory diagnoses of the three main infectious etiologies of VV. The clinical diagnosis of *Candida* VV was based on the finding of a white vaginal discharge, pH of less than 4.5, negative whiff test and – essential to the diagnosis – the findings in wet mount of budding hyphae with or without the presence of spores, while the laboratory diagnosis of *Candida* infection rested on the finding of *Candida* strains on culture.

The clinical diagnosis of *Trichomonas* infection was based on the finding of a yellow or greenish vaginal discharge with a pH of 4.5 or higher, and – essential and sufficient to the diagnosis – a wet mount showing mobile protozoa and an increased number of leukocytes. The laboratory diagnosis of *Trichomonas vaginalis* rested on the growth of *Trichomonas* in Diamond agar.

**Table 1** Criteria of clinical and laboratory diagnosis

| *Etiology of vulvovaginitis* | *Clinical diagnosis* | *Laboratory diagnosis* |
|---|---|---|
| *Candida* vulvovaginitis | White discharge, vulvar pruritus, pH < 4.5, whiff test (–), hyphae (+) on wet-mount microscopy* | Growth in culture |
| Bacterial vaginosis | Gray–white homogeneous discharge, pH ≥ 4.5, whiff test (+), clue cells > 20% in wet-mount microscopy** | Gram stain |
| *Trichomonas* vaginitis | Yellow-green discharge, pH ≥ 4.5, wet-mount – motile trichomonads*, leukocytes in smear | Growth in Diamond agar |

*Criterion essential and sufficient for diagnosis; **documentation of at least three of the four criteria necessary for diagnosis

The clinical diagnosis of bacterial vaginosis (BV) was based on the Amsel's criteria[7], i.e. the presence of at least three of the following: a homogeneous gray–white vaginal discharge, pH of 4.5 or higher, positive whiff test and wet mount showing clue cells in more than 20% of epithelial cells. The laboratory diagnosis of BV was based on the finding of clue cells in the Gram stain.

If the essential criteria for two infections – for example *Candida* and *Trichomonas* – were clinically documented, a diagnosis of 'mixed infection' was made.

Excluded from the study were women in whom gonorrheal or chlamydial infection was detected in the endocervical specimens, or in whom pelvic inflammatory disease, condylomata acuminata, herpes genitalis or urinary tract infection was diagnosed during the examinations. Other exclusion criteria were pregnancy or breastfeeding, a significant medical disease such as diabetes mellitus or renal failure, antibiotic treatment and corticosteroid or immunosuppressive medication in the previous 3 weeks. Women using an intrauterine contraceptive device (IUCD) were not enrolled since the vaginal discharge sometimes associated with IUCDs could have affected the evaluation of the discharge. No women currently menstruating were enrolled into the study. The clinical diagnoses as well as each symptom and sign were correlated with the laboratory diagnosis by measurement of agreement of $\kappa$. In addition, social and demographic parameters were compared between groups of patients with the various laboratory diagnoses. Differences between the groups were examined for significance using the *t* test for continuous parameters with a normal distribution, and $\chi^2$ and Fisher's exact tests for categorical parameters. Positive predictive values, sensitivity, specificity and odds ratio with 95% confidence intervals (CI) of the bedside tests were calculated.

### RESULTS

One hundred and fifty-three women with vaginal discharge with a clinical diagnosis of VV of one of the following infectious etiologies – *Candida*, *Trichomonas* or BV – participated in this study. The laboratory tests revealed that 55 (35.9%) of the women with a clinical diagnosis of VV in fact had normal flora, and the other 98 (64.1%) did indeed suffer from infectious vulvovaginitis, the principal infectious micro-organism being *Candida* species (Table 2). Of 68 patients with a positive *Candida* culture, 61 had a clinical diagnosis of candidiasis. *Candida* non-albicans was diagnosed in 10.3% of all candidal infections (data not tabulated). Bacterial vaginosis was less frequent, and *Trichomonas* and mixed infection were diagnosed only rarely. Table 2 also depicts a poor agreement ($\kappa = 0.18$) between the gynecologist's clinical diagnosis of candidal infection and the definitive laboratory diagnosis, as initially 115 of the enrolled women (75.2%) were diagnosed clinically as having candidal infection. The clinical diagnosis of *Candida* was verified in only 53% of the women, while 37.4% were shown to have normal flora.

Of the 26 women diagnosed clinically as having BV, the diagnosis was verified by Gram stain in only 6 (23.1%). The gynecologist correctly diagnosed only one patient of the six where *Trichomonas* grew in Diamond medium.

**Table 2** Comparison of the clinical and laboratory diagnoses. $\kappa$ agreement = 18%; numbers in parentheses are percentages of cases in the same column

| | *Clinical diagnosis (n (%))* | | | | |
|---|---|---|---|---|---|
| *Laboratory diagnosis* | Candida | *Bacterial vaginosis* | *Trichomoniasis* | *Mixed infection* | *Total* |
| Normal flora | 43 (37.4) | 9 (34.6) | 0 | 3 (30) | 55 |
| *Candida* species | 61 (53) | 6 (23.1) | 0 | 1 (10) | 68 |
| Bacterial vaginosis | 6 (5.2) | 6 (23.1) | 1 (50) | 2 (20) | 15 |
| Trichomoniasis | 0 | 4 (15.4) | 1 (50) | 1 (10) | 6 |
| Mixed infection | 5 (4.3) | 1 (3.8) | 0 | 3 (30) | 9 |
| Total | 115 | 26 | 2 | 10 | 153 |

There was a non-significant trend for women with a laboratory diagnosis of infectious vaginitis to be younger than those with normal flora and there was no correlation between the marital status of the patients and the specific causes of infection (Table 3).

Table 4 shows a non-significant trend of a lower rate of pruritus in women with normal flora compared to women with infectious vulvovaginitis. The amount of vaginal discharge was greater (according to the physician's estimate) in VVC than in BV or in the presence of normal flora, although not to a significant extent. There was also a trend to having a white-colored discharge in cases of *Candida* or mixed flora, while *Trichomonas* or BV was characterized as being gray in color (data not tabulated).

Tables 5 and 6 depict a significant association between pH of the discharge and the infectious vaginal agent. *Candida* infection was associated with pH levels of lower than 4.5 ($p < 0.0001$, odds ratio = 5.0, 95% CI: 2.46–10.16, positive predictive value 68.4%), while only a few cases with BV or *Trichomonas* were associated with a pH of 4.5 or lower. The whiff test was positive in only a small percentage of BV cases, similar to the findings in *Candida* and normal flora ($p$ = NS). Clue cells were documented by the gynecologist in 53.3% of patients with a laboratory diagnosis of BV ($p < 0.02$, odds ratio = 6.02, 95% CI: 1.98–18.32, positive predictive value 26.7%), and in 66.7% of smears of those with a laboratory diagnosis of *Trichomonas* infection. The physician had determined that hyphae existed in 75% of women finally shown to have *Candida* infection ($p = 0.048$, odds ratio = 1.99, 95% CI = 1.00–4.02), but also in 60% of women with normal flora and in 60% of those with BV. In the KOH smear test only a few more hyphae were seen than in the saline smear (data not tabulated).

**Table 3** Demographic characteristics

| *Laboratory diagnosis* | *No. of women (n (%))* | *Age (mean (SD))* | *Age (range)* | *% married* |
|---|---|---|---|---|
| Normal flora | 55 (35.9) | 32.8 (11) | 18–55 | 56.4 |
| *Candida* species | 68 (44.4) | 31.5 (8.3) | 19–53 | 61.8 |
| Bacterial vaginosis | 15 (9.8) | 30.7 (6.9) | 22–45 | 60 |
| Trichomoniasis | 6 (3.9) | 25.7 (7.4) | 18–37 | 66.7 |
| Mixed infection | 9 (5.9) | 29.8 (9.3) | 18–46 | 66.7 |
| Total | 153 | | | |

**Table 4** Correlation of symptoms with laboratory diagnosis; $p \leq 0.1$; numbers in parentheses are percentages of cases in the same row

| | *Symptom (n (%))* | | | |
|---|---|---|---|---|
| *Laboratory diagnosis* | *Increased discharge* | *Increased discharge and pruritus* | *Pruritus alone* | *Total* |
| Normal flora | 23 (41.8) | 32 (58.2) | – | 55 |
| *Candida* species | 21 (30.9) | 46 (67.6) | 1 (1.5) | 68 |
| Bacterial vaginosis | 2 (13.3) | 13 (86.7) | – | 15 |
| Trichomoniasis | 1 (16.7) | 5 (83.3) | – | 6 |
| Mixed infection | 2 (22.2) | 7 (77.8) | – | 9 |
| Total | 49 (32.0) | 103 (67.3) | 1 (0.7) | 153 |

### DISCUSSION

The main finding of this study is that in only 66.1% of women with a clinical diagnosis of VV was a laboratory diagnosis of infection established. Most inaccuracies occurred with BV, where only 23.1% of the clinical diagnoses were confirmed by Gram stain. VVC was confirmed by culture in only 53.5% of those with clinical diagnoses of VVC. Hyphae were documented by the gynecologists in cases that were later diagnosed by the laboratory with normal flora or with BV. These unexpected findings cast doubt on the expertise of the gynecologists participating in the study, and we presumed that their experience with the use of microscopy and the other bedside tests was inadequate. However, the same rate of discrepancies between the clinical and laboratory diagnoses was found regardless of the examiner. It should also be taken into account that the same or an even higher rate of mistakes in clinical diagnoses is possibly made every day by many gynecologists. Therefore, we suggest that the common practice of relying on the clinical diagnosis for establishing the treatment may be unreliable, and needs to be re-evaluated.

Another possible explanation for the findings in the present study of a poor correlation between clinical and laboratory diagnosis is that the laboratory rather than the clinical diagnoses were

**Table 5** Correlation of signs with laboratory diagnosis; numbers in parentheses are percent of cases in the same column. Some women had more than one sign/test

| | Laboratory diagnosis (n (%)) | | | | |
|---|---|---|---|---|---|
| *Sign/test* | *Normal flora* | *Candida* | *Bacterial vaginosis* | *Trichomoniasis* | *Mixed infection* |
| Excessive discharge | 12 (21.8) | 25 (36.8) | 1 (6.7) | 3 (90) | 2 (22.2) |
| pH < 4.5 | 17 (30.9) | 39 (57.4)* | 1 (6.7) | 0 | 0 |
| Positive whiff test | 4 (7.3) | 4 (5.9) | 1 (6.7) | 3 (50) | 1 (11) |
| Clue cells > 20% | 0 | 0 | 8 (53.3) | 4 (66.7) | 0 |
| Positive hyphae (10% KOH) | 33 (60) | 51 (75) | 9 (60) | 1 (16.7) | 8 (88.9) |
| Total | 55 | 68 | 15 | 6 | 9 |

*$p < 0.0001$ (positive predictive value: 68.4%)

**Table 6** Reliability of diagnostic tests

| *Test* | *Diagnosis* | *p value* | *% sensitivity* | *% specificity* | *Positive predictive value* | *Odds ratio* | *95% CI* |
|---|---|---|---|---|---|---|---|
| pH < 4.5 | VVC | < 0.0001 | 57.4 | 78.8 | 68.4 | 5.00 | 2.46–10.16 |
| Clue cells > 20% | BV | < 0.02 | 53.3 | 84.2 | 26.7 | 6.02 | 1.98–18.32 |
| Hyphae | VVC | < 0.048 | 75 | 40 | 50 | 1.99 | 1.00–4.02 |
| Positive whiff test | BV | NS | N/A | N/A | 7.7 | N/A | N/A |

BV, bacterial vaginosis; CI, confidence interval; N/A, not applicable; NS, not significant; VVC, vulvovaginal candidiasis inaccurate. Indeed, most patients with a positive *Candida* culture had had a clinical diagnosis of candidiasis. Possibly, some of the infectious micro-organisms failed to grow in culture. A possible inhibitor of culture growth may have been the patient's use of an anti-fungal or anti-trichomonal agent prior to obtaining the culture. Although in the present study patients were enrolled only if they had not taken anti-fungal or anti-trichomonal medications for a month, we suspect that some of the women had a supply of anti-fungal or anti-trichomonal cream or vaginal pessaries left from previous VV episodes. They may have inserted a vaginal suppository or applied a cream to ease the symptoms, and then come to the doctor's office only when this self-treatment failed to completely alleviate their symptoms. These treatments could have inhibited the growth in culture of the *Candida*.

In addition it is difficult to ascribe a 'gold standard' in the laboratory diagnosis of these infections. It could be argued that the polymerase chain reaction (PCR) test may be more sensitive than culture for identification of *Candida* species and *Trichomonas*. However, PCR has not yet been introduced into routine clinical use in cases of VV. Delay in cultivating the specimens is another possible cause of lack of culture growth of *Candida*. In the present study efforts were made to transfer the specimens to the laboratory immediately after collection. In addition, experienced personnel examined the cultures and stained slides. We therefore argue that the laboratory diagnosis in these cases could serve as the gold standard. Hence, based on bedside tests, the clinicians over-diagnose patients with vaginal discharge as suffering from infectious vulvovaginitis.

BV infection was detected in only 15 women and *Trichomonas vaginalis* in six. These small numbers limit discussion of the findings. However, the discrepancy between the clinical and laboratory diagnoses of BV is particularly disturbing, but could have resulted from the clinical nature of the diagnosis. Clinical assessment is traditionally used for diagnosis[7,8]. However, in the present study, which based the diagnosis of BV on Amsel's criteria (pH ≥ 4.5, homogenous discharge, clue cells and a positive whiff test)[7] – which might suffice for the diagnosis of BV – nevertheless at the same time some of them could have represented a candidal infection. In addition, a mixed infection of VVC and BV might have been responsible for another interesting finding, that of many women with a laboratory diagnosis of BV complaining of pruritus – a symptom not generally associated with pure BV.

Based on the laboratory diagnosis, the most common vaginitis was VVC, with a frequency of 35.9%. The incidence of BV was only 9.8%, which is not in accord with the situation in other countries, such as the USA, where BV is the most common VV.

From Tables 4–6 it seems that the only symptom that might be considered reliable in establishing the infectious etiology of VV is pruritus combined with discharge, which was associated with VVC in 67.6% of cases and with mixed infection (containing VVC) in 77.8%. The only useful bedside test was a pH lower than 4.5, which carried a positive predictive value of 68.4% and odds ratio of 5.00 (95% CI: 2.46–10.16) of predicting a candidal infection. Nevertheless, a significant number of women with normal vaginal flora had a pH of the vagina between 3.8 and 4.5. The other classic sign, hyphae representing VVC, had an odds ratio of 1.99 (95% CI: 1.00–4.02) for detecting candidiasis. A positive whiff test failed as a sign of BV, since it did not differentiate between VVC, BV, *Trichomonas* infection and normal flora.

There may be several causes for the low specificity and sensitivity of most of the bedside tests. In point of fact, the reliability of these bedside tests to make the diagnosis of infectious VV has never been established. Early studies indicated that each symptom and sign was associated with a range of diagnoses[8,9]. For example, some *Candida* infections were associated with a pH of 5–5.9 and also with pH of 6–7.2, while BV has been found to be associated with pH lower than 4.1[8,9]. Determination of pH may be biased by the lack of uniformity in obtaining the test – for example, taking the sample for pH from different locations in the vagina.

Although these tests are not reliable, they appear in textbooks and review papers[10], as aids for the practicing clinician to establish a clear-cut diagnosis in each case of VV and to help him or her prescribe the proper treatment. Even in our study, in 60% of cases with a laboratory diagnosis of normal flora, Hyphae were documented in wet mount microscopy. Therefore, although most textbooks continue to emphasize the unequivocal significance of the bedside tests for the diagnosis of VV, our study indicates that vaginal cultures should be used for the diagnosis of VVC and trichomoniasis.

### CONCLUSIONS

Based on these findings, the current approach to the diagnosis of VV should be reconsidered. The classical symptoms and signs were proven not to be reliable diagnostic measures, while being time-consuming. Obviously, to target treatment a proper diagnosis has to be made. Therefore cultures and Gram stains by the laboratory should be made in each case of VV, or, alternatively, a standard treatment for all VV, covering all possible infectious micro-organisms, should be developed.

### REFERENCES

1. Thomason JL, Gelbart SM, Broekhuizen FF. Office and clinical laboratory diagnosis of vulvovaginal infections: an overview. In Horowitz B, Mardh PA, eds. *Vaginitis and Vaginosis*. New York: Wiley-Liss Inc., 1991:93–108
2. Sobel JD, Faro S, Force RW, *et al.* Vulvovaginal candidiasis: epidemiologic, diagnostic and therapeutic considerations. *Am J Obstet Gynecol* 1998; 178:203–11
3. Kaufman RH. Establishing a correct diagnosis of vulvovaginal infection. *Am J Obstet Gynecol* 1988; 158:986–8
4. Nyirjesy P, Seeney SM, Terry Grody MH, *et al.* Chronic fungal vaginitis: the value of cultures. *Am J Obstet Gynecol* 1995;173:820–3
5. Gelbart SM, Thomason JL, Osypowski PJ, *et al.* Culturing *Trichomonas vaginalis*. In Horowitz B, Mardh PA, eds. *Vaginitis and Vaginosis*. New York: Wiley-Liss Inc., 1991:109–14
6. Lossick JG. The diagnosis of vaginal trichomoniasis. *JAMA* 1998;259:1230
7. Amsel R, Totten PA, Spiegel CA, *et al.* Nonspecific vaginitis: diagnostic criteria and microbial and epidemiological association. *Am J Med* 1983; 74:14–22

8. Holst E. Bacterial vaginosis: clinical and microbiological findings. In Horowitz B, Mardh PA, eds. *Vaginitis and Vaginosis*. New York: Wiley-Liss Inc., 1991; 115–20
9. Hilier SL. Laboratory diagnosis of yeast vaginitis. In Horowitz B, Mardh PA, eds. *Vaginitis and Vaginosis*. New York: Wiley-Liss Inc., 1991;121–4
10. Friedrich EG. Vaginitis. *Am J Obstet Gynecol* 1985; 152:247–51

RECEIVED 11/30/01; ACCEPTED 04/03/01
